# Extended Abstracts: International Symposium on Immunopathogenesis of Chlamydia Infections

**DOI:** 10.1155/S1064744996000373

**Published:** 1996


Infectious Diseases in Obstetrics and Gynecology 4:176–193 (1996)

© 1996 Wiley-Liss, Inc.

### International Symposium on Immunopathogenesis of Chlamydia Infections
### December 6-8, 1996
### Cornell University Medical College
### 1300 York Avenue, New York, NY
### USA

### Extended Abstracts

Received September 15, 1996
Accepted October 1, 1996

### Human Studies on the Association of Cellular Immune Factors in Trachoma With Infection and Disease.

***LD Bobo*, NG Novak, S Vitale. H Mkocha, Y-H Hsieh, B Munoz, S West, TC Quinn***

*Infectious Diseases and Dana Center, Johns Hopkins University, Baltimore MD*

### Introduction

Trachoma, which is caused by *Clamydia trachomatis*, is the most common cause of infectious blindness in the world, with cases predicted to exceed 12 million within the next 30 years (1). Most children living in Tanzanian villages where trachoma is endemic experience chlamydia ocular infection with prevalence ranging from 40-60%. In general, infection prevalence is lower in adults, but the severe scarring sequelae, trichiasis, is more prevalent in women suggesting a higher infection rate. A delayed-type hypersensitivity response to reinfection is thought to participate in the development of pathology, however the underlying mechanism is not clear (2). Although chlamydia infection prevalence is higher in children, we have identified subgroups which have either severe disease in the absence of risk factors, such as, unclean faces, versus those who clear infection with minimal disease. It is thus important to compare immunogenetic as well as socio-environmental differences between people who differ in their ability to clear infection and in their pathologic response to infection.

Trachoma is a useful model for these comparisons. Clinical scoring of disease severity and ocular samples are easily obtained by field personnel without using invasive procedures. These attributes would be useful in longitudinal studies with serial samplings for determining a specific immune response associated with onset and clearance of infections or development and resolution of disease. Because of the important immunoregulatory and pathogenic role of cytokines in other infectious diseases the following studies deal with the local cytokine response associated with *C. trachomatis* ocular infection and disease severity. In addition, we discuss the diversity of *omp*-1 genovariants in our study population as suggestive that other immunointervention strategies should be considered.

### Cytokine Studies

Using cross-sectionally obtained samples, we compared the types and levels of cytokine mRNA from conjuctival cells and of cytokine protein from tears with infection status and disease presentation. We chose to measure those mediators which are involved in the Thl/Th2 paradigm, or which are known to be proinflammatory or cicatrogenic. The population for the mRNA study was composed of 50 adults and children living in trachoma-endemic villages in central Tanzania. Disease was scored using the WHO grading scheme of trachomatous inflammation obscuring at least 50% of tarsal vessels (TI), the presence of 5 follicles (TF), TI and any scarring (TIC), scarring only (TC), and no disease signs in either eye (NS). Chlamydial DNA was detected by PCR-EIA (3), and mRNA was measured using RT-PCR-EIA with amplified DNA levels expressed as log fluorescence units (4). Sample cDNAs were standardized to each other according to β-actin level.

As in previous studies, we found that chlamydial DNA load was higher in the presence of inflammation: TI, 2.31 ± 0.35; TF, 1.61 ± 0.18; TIC, 2.02 ± 0.24; TC, 1.41 ± 0.22; and NS, 0.8 ± 0.11. In Figure infection status is compared to transcripts levels, with boxes encompassing 75% of the data, and medians indicated by a horizontal line within the box.

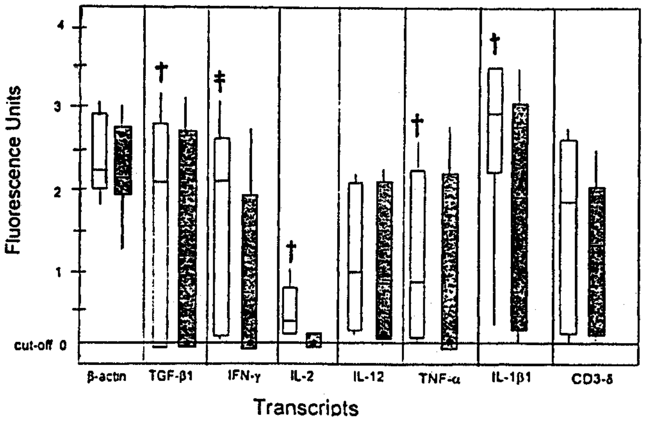

FIGURE 1 (modified) (4).

An asterisk at the bottom of the box indicates that the median was at the lowest point of the box. Chlamydia positivity is indicated with a clear box, and a gray box indicates no chlamydia. A (†) indicates a level of significance of $p < 0.05$, whereas (‡) indicates $p < 0.001$.

In Figure 2, clinical disease score is compared to transcripts and the same conventions are use as in Figure 1. Symbols for disease score are included in Figure 2. TGF-β1, TNF-α, and IFN-γ were significantly elevated in the TI group ($p < 0.001$), as well as for TF compared to NS ($p < 0.05$). TGF-β1 was significantly elevated ($p< 0.05$) for TIC or TC compared to NS. IL-2 and IL-12p40 were significantly elevated for TI ($p < 0.05$). IL-12p40 was significantly associated with IFN-γ ($p < 0.003$). Although IL-12p40 was not significantly associated with chlamydia infection (6/10 with chlamydia and IL-12), all 10 people were in the TI/TF category. IL-2 was more likely to occur with IFN-γ and IL-12 ($p < 0.001$). IL-4,5 and 10 transcripts were not found in any samples, and IL-6 mRNA was detected in only 2 people with TI. Elevated ICAM-1 (not shown), was highly associated with infection and TI ($p < 0.05$). CD3-δ T cell receptor transcripts were elevated in all disease categories as compared to NS.

Tear cytokines from 36 children were measured by ELISA. Eighteen children had chlamydia infection by PCR-

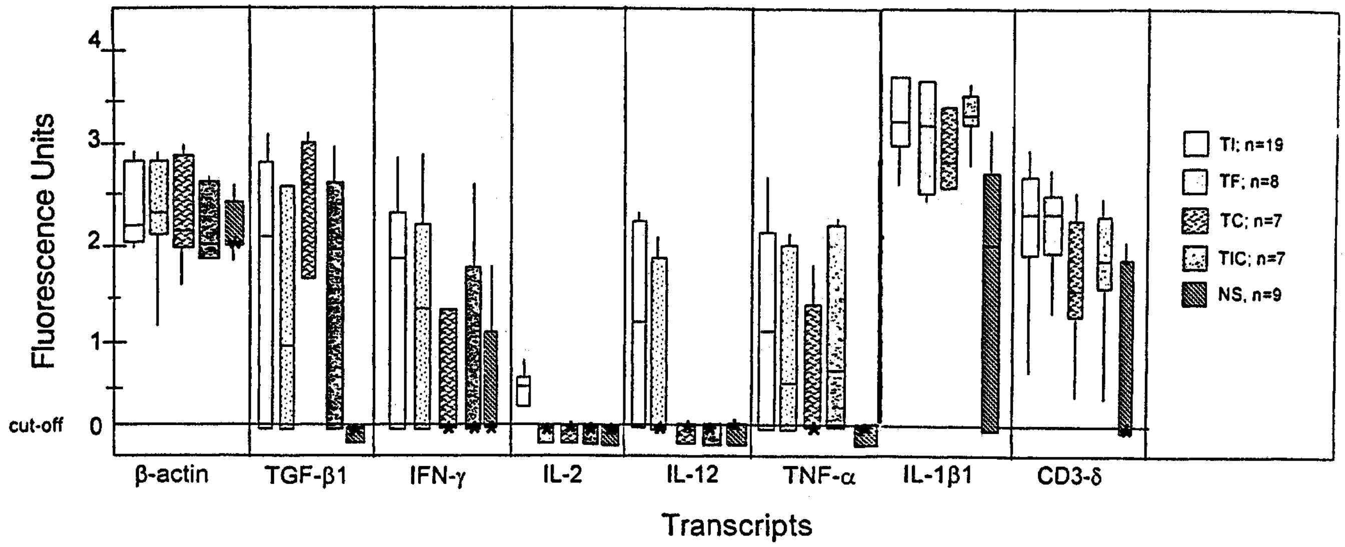

Figure 2

EIA. There were 12 in each clinical category of TI, TF, or NS with 6 chlamydia-positive and 6 chlamydia-negative. There was a significant association of IL-1β and IL-6 with TI (p <0.001) regardless of infection status, but mean levels (pg/ml) for IL-1β were higher in the presence of chlamydia infection (7.5 IFN-± 1.24 compared to 2.5-±1.51). Only one child with NS had IL-1β and IL-6 but also had chlamydia. TNF-α was found in 3 people with TI, and IFN-γ in only 2 persons who had TI and chlamydia. IL-12p40 was found in 50% of people with TI or TF, regardless of infection status. IL-4 was not detected. IL-2, IL-10, and TGF-β1 were not measured.

In summary, cytokine mRNA levels were higher when chlamydia was present as was IL-1β protein. Highest chlamydial DNA levels were associated with inflammation (TI and TIC). The cytokine response was predominantly pro-inflammatory with respect to IL-1β, IL-6, TNF-α, IFN-γ, and TGF-β1. Some mediators of the Th1 response (IL-2 and IL-12p40) were only associated with the earlier disease presentations of TI or TF, but not with scarring. TGF-β1, which is important in wound healing but can induce pathogenic scarring, was elevated in the presence of infectious sequelae TC and TIC. TNF-α levels were most highly associated with TI, TF, and TIC with chlamydia positivity. We were unable to define the role of IFN-γ in protection or persistence since IFN-γ was occasionally present with NS and chlamydial DNA. The finding of higher IFN-γ and TNF-α with low IL-2 indicates that natural killer cells should be considered as a potentially important source of these cytokines. Although CD3-δ transcripts were elevated in disease states, we did not detect the Th2 mediator, IL-4, by any method. On the other hand, another Th2 effector, IL-6 protein, was highly associated with TI/TF, whereas mRNA was infrequently found. Due to the cross-sectional nature of these studies, we were not able to say with certainty if a particular cytokine pattern was characteristic of resolution of infection or protection from disease.

### Longitudinal Study of Infection Duration

One purpose of this study was to determine if infection duration varies between children who have similar socio-environmental risk factors. The study included 54 children aged 1-12 years from one village in Tanzania who had clinical disease scores and conjuctival swabs taken weekly for 3 months (n=632). This set included 12 families with 30 siblings. Persistent chlamydial DNA by ligase chain reaction (LCR) was found for 10-12 weeks in 37% (20/54), with 19/21 infected on week 12. Two children were infected on weeks 11-12 and 6-12. One child was infected at weeks 3,5, and 7-9. Eleven children (20.4%) were infected at one or 2 week intervals during the 12 weeks. The remaining 18 (33.3%) did not have chlamydia infection. In terms of related individuals, all siblings were persistently infected in 25% of families, in another 25% one sibling was persistently infected with the others sporadically or uninfected, and 25% had one sib sporadically infected with the other sibs sporadically or not infected.

As in earlier studies, chlamydia positivity was higher in the TI group (62.7%) compared to TF (42.6%) and NS (10.4%). In addition, the chlamydial load was higher in association with TI (2.29 ± 0.35), and NS (0.51 ±0.12) as expressed by the LCR ratio (positives are ≥ 1). Children who were persistently infected generally had high chlamydial loads ( >2) in association with TI, whereas sporadically infected children usually had LCR ratios of between 1-2 and had NS. Neither gender nor a particular age grouping were significantly associated with increased chlamydial infection or disease presentation although there was a trend for more females to be infected p = 0.142 (OR = 0.41, 95%

CI, 0.33-28.44).

In summary, this data indicates that 1) infection duration subgroups could be identified as "persistent" or "sporadic"; 2) due to the effects of truncation we could not determine the true duration of infection but that approximately 40% may have been persistently infected beyond 12 weeks; 3) siblings did not necessarily share the same infection pattern; 4) chlamydial load was highest in children who were in the "persistent" infection group; 5) there was a trend for more females to be infected than males, but the difference was not statistically significant.

### *OMP*-1 Sequencing Studies

The purposes of these studies were to determine 1) what the diversity of ocular *omp*-1 genovariants in central Tanzanian villages was from 1986-1995, 2) if there was any correlation of a genovariant with infection duration of disease severity, and 3) if the same person sampled at 2 time points (1989 and 1995) had the same genovariant. We sequenced amplified *omp* -1 chlamydial DNA scraped from DFA slides made from conjuctival samples of 74 children dispersed among 9 villages in 1986 and compared sequences to reference strains. In summary, Tanzanian villages had a diversity of genovariants; serovar A = 19; B = 7; Ba = 3; C = 1; D = 2, E = 2; and F = 1. The diversity in any village ranged form 3-9 genovariants. Both mixed genovar (26%) and mixed serovar (2.7%) infection (A/E, A/F) were found . Genovariants possessed "signature" substitutions in VS1 and 2, and 2.7% had amino acid substitutions in known B cell neutralizing epitopes. For 9 children who were sampled in 1989 and 1995, 6 had the same serovar at 2 time points but only 2 had the same genovariant, and mixed serovar infection occurred (A/B). In the previous "Longitudinal Study of Infection Duration," persistently infected children had the same genovariant throughout as did those who were intermittently infected. Children within a family had the same genovariant. In addition, there was no particular association of a genovariant with infection duration. Finally, we have not found a significant association of an *omp*-1 genovariant with disease severity in any of our studies.

### Conclusions

We have presented the results of substudies dealing with local cellular immune response, infection duration, and *omp*-1 DNA sequencing of *C. trachomatis* from people living in trachoma endemic communities. We feel that this data is consistent with the hypothesis that there are some people who are persistently infected or get reinfected continuously, and that they characteristically have higher *C. trachomatis* burden and suffer more intense disease. In addition, since the presence of chlamydial organisms is associated with the highest levels of certain inflammatory and cicatrogenic cytokines, the sequelae of severe scarring and trichiasis are likely to occur as a result. Chlamydia-associated components which stimulate harmful cellular mediators are likely to be common to all *C. trachomatis.* strains, such as heat shock protein or another candidate. It is not known whether cross-stimulation of the immune system by chlamydial hsp and human hsp could amplify the effects of infection in some hosts. The inability to clear infection of the occurrence of intermittent re-infection (separated by weeks or years) with the same genovariant suggests that the cytokine response to chlamydia infection could be inappropriate for maintenance of immunity, and that some other additional factor in a successful host determines rapid clearance and protection from disease. To gain a clearer understanding of what controls chlamydial clearance, we will compare socio-environmental risk factors with infection duration and disease intensity subgroups in families (mothers and siblings), and in women with trichiasis with local cytokine and peripheral mononuclear cell response to chlamydial antigens and with HLA haplotype.

### References

1. Schachter J and CR Dawson . 1990 Scand Infec Dis 69:55.
2. Morrison RP, RJ Bellad, K Lyng, HD Caldwell: 1989 J Exp Med 170:1271-1283.
3. Bobo L, B Munoz, R Viscidi, T Quinn, H Mkocha, W. West: 1996 Infec Immun 64:3273-3279.

### Antigen and Isotype Specificity of Human B Cell Responses in Immunopathogenesis of Genital *Chlamydia trachomatis* Infection

***S Ghaem-Maghami, PE Hay, DJM Lewis***
*St George's Hospital Medical School, London, UK*

Mucosal infection with *Chlamydia trachomatis (C. trachomatis)* is the most common sexually transmitted disease and a cause of infertility, ectopic pregnancy, and perinatal morbidity.

### Objectives

To characterise *Chlamydia trachomatis*-specific B cell response to human genital infection, in terms of relative magnitude of antibody class, kinetics of response and immunodominance of chlamydial antigens at different stages of disease and treatment.

### Methods

We have employed the cell based enzyme linked immunospot assay (ELISPOT) to characterise trafficking B cell response during human genital chlamydial infections by detecting antibody secreting cells (ASCs) spontaneously secreting IgG, IgA, and IgM antibodies against *C. trachomatis* antigens. We have studied a total of 36 men and 20 women treated for chlamydial infection and 23 controls (healthy asymptomatic men and women). No ASCs have been seen in any of the 23 controls. In keeping with "mucosal compartmentalisation," IgA was the predominant isotype secreted by trafficking anti-EB ASCs in men with uncomplicated non gonococcal urethritis (NGU) and women with cervicitis (Fig. 1). However, in subjects with extragenital manifestations (arthritis/conjunctivitis) there was an appar-

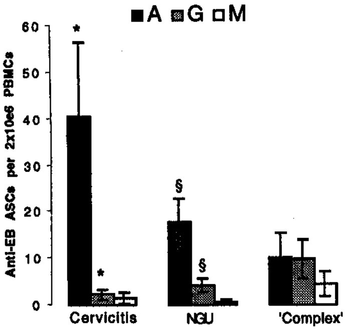

Fig 1: Frequency of anti-EB antibody class of circulating ASCs in women with cervicitis (n=20), and men with NGU (n=36) or NGU plus arthritis and/or conjunctivitis ('complex', n=6). Errors bars SEM *IgA>IgG $p$=0.01, §IgA>IgG $p$=0.02

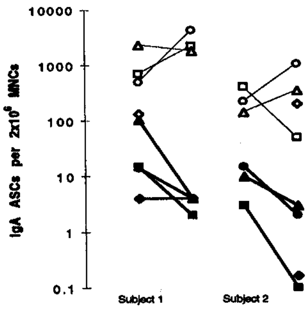

Fig 2: Chlamydia specific IgA ASCs in blood (closed symbols) and cervix (open symbols) of 2 women with chlamydial cervicitis before and 6 weeks after treatment. ●EB, ◆hsp60, ■ MOMP, ▲ pgp3.

ent break down in the compartment and a relatively increased IgG ASC response and decreased IgA ASC response.

### Results

By employing ELISPOT and using whole Chlamydia organisms, recombinant proteins and separated antigens, we have characterized kinetics and antigenic immunodominance of response. We have studied 25 patients with whole chlamydia elementary bodies and recombinant hsp60, MOMP, and pgp3 and been able to illustrate relative antigen immunodominance between IgG and IgA. There is no association between IgA ASC response to hsp60 and immunopathology but IgG ASC response to this antigen needs to be investigated in patients with "complex" disease as preliminary results indicate a relative switch to IgG ASC response to hsp60 in these patients.

We have characterised the kinetics of the response at different times after presentation, when two weeks antibiotic treatment is commenced. Although patients were at different stages at presentation, peak trafficking ASCs occur around 1-2 weeks after treatment, disappearing from the circulation 3-4 weeks later. Mucosal B cells have been directly studied using cervical biopsies and urine and results compared with blood. Trafficking IgA ASCs are far less frequent than in the mucosa but the pattern of antigenic specificity is very similar suggesting homing of ASCs to mucosal sites. (Fig. 2).

### Conclusion

IgA is the predominant antibody produced in response to an uncomplicated chlamydial genital infection with a relative switch to systemic IgG activation when there are extra-genital manifestations of the infection. There is a rise in the number of ASCs immediately following treatment suggesting increased antigen presentation. The circulating B cell response to Chlamydia infection is tightly controlled, disappearing from the circulation a few weeks after treatment indicating that the presence of Chlamydia-specific B cells in the circulation represents acute infection. Using the ELISPOT technique it is possible to define B cellular immune responses to chlamydial antigens in patients with different manifestations of chlamydial diseases. This will clarify the role of these antigens at different stages of disease and its complications.

Such data on the characterisation of human B cellular mucosal/systemic responses to *C. trachomatis* and their evolution into disease pathogenesis is a useful model of genital mucosal systemic infection, with implications for understanding immunopathology and for delivery of novel human vaccines.

---

### Repetitive Extragenic Palindromic Sequence Polymorphism Based Polymerase Chain Reaction (rep-PCR) is a Useful Tool in Diagnostics of *Chlamydia psittaci* Infection in Sheep and in Studies of Field and Vaccinial Chlamydial Strains

***SP Martinov[1], E Tcherneva[1], B Jersek[2]***

*[1]Central Veterinary Research Institute, Sofia, Bulgaria,*

*[2]Biotechnological Faculty of University of Ljubljana, Slovenia*

The aim of the present work is to prepare and test vaccines against ovine chlamydial abortion and the investigation of vaccinial and field strains of *Chlamydia psittaci* of different origin by rep-PCR.

During the last ten years three inactivated vaccines PM-1, PM-2, and PM-3 were prepared against chlamydial abortion in sheep (1,2,3). The methods employed included: chicken embryos, cell cultures, electron microscopy (EM), mechanical chomogenization, purification, formaldehyde inactivation, aluminum hydroxide adsorption, sonication, complement fixation test (CFT), enzyme immunoassay (ELISA), serum neutralization assay (SNA), polyacrylamide gel electrophoresis (PAGE), Western blot, checking for sterility and immunogenicity by challenge with live Chlamydiae at 85 day of gestation. Strains GV, P, Kt, D (vaccines PM-1 and PM-2) and A22 and S26/3 (vaccine PM-3) of *C. psittaci*. psittaci var.ovis were used and all the stages of the vaccine preparation were checked by EM of negatively stained suspensions (Fig. 1). The vaccines were concentrated and purified. They possessed a high content of chlamydial antigen measured by CFT and ELISA (5885 IFU/ml at 1:50 dilution of PM-3).

The major outer membrane protein (MOMP) was revealed in the purified chlamydial suspensions by PAGE-immunoblotting (Fig.2, pattern marked with arrow). The vaccines were areactogenic and completely innocuous for guinea pigs and sheep. They induced considerable serological response (CFT, ELISA, SNA) for at least 10 months after single or double immunization with 0.75-1.0 ml s.c. Table 1 shows a very good response with CFT. The results from the SNA performed with sera from sheep vaccinated with PM-3 are presented in Table 2. It is seen that the level of reduction of the infectiousness determined by the sera, grows during the second month. Between months 2 and 3 this percentage (i.e., the level of immunity) decreases and it grows up again at the beginning of month 4, and it preserves a stable high level (months 4 and 5). Generally the vaccine produced a good immune response checked by SNA. The challenge experiment consisted of a single immunization

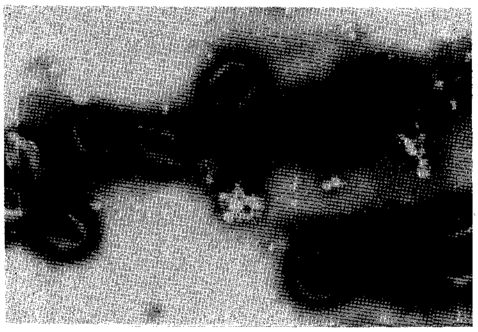

Figure 1

of 10 pregnant ewes with PM-3 vaccine and challenging them and 19 unvaccinated controls more with a two-strain innoculum (A22 and S26/3) containing $10^6$ $ELD_{50}$. Table 3 presents the findings in regard to lambing and abortions. None of the vaccinated ewes aborted. Only one placenta appeared macroscopically abnormal and only two sheep yielded Chlamidiae from the placenta. In contrast there were 11 abortions and 16 dead animals from 28 in the control group. 15 of all the placentas tested were gravely affected and in 15 of the animals chlamydial excretions were found.

Table 1. Chlamydial CF-Antibodies in the Blood Sera of Sheep After Immunization with Vaccine PM-3

| Term[a] | S.I.[b] | CFT (+) | MGT | D.I.[c] | CFT (+) | MGT | MGT[d] |
|---|---|---|---|---|---|---|---|
| 1.5 | 14 | 14/100 | 17.1 | 13 | 13/100 | 18.4 | 17.8 |
| 2.5 | 14 | 14/100 | 64 | 13 | 13/100 | 60 | 64 |
| 3 | 14 | 14/100 | 112 | 13 | 13/100 | 94 | 104 |
| 3.5 | 14 | 14/100 | 120 | 13 | 13/100 | 94 | 104 |
| 4 | 14 | 14/100 | 69 | 13 | 13/100 | 74 | 69 |
| 7 | 14 | 12/85.7 | 13.9 | 13 | 11/84.6 | 19.2 | 11.3 |
| 10 | 14 | 11/78.5 | 4.6 | 13 | 8/61.5 | 3.2 | 4.0 |

[a] Term of testing in months.
[b] Single immunization.
[c] Double immunization.
[d] MGT; management for both groups.

The experiment with the animals used in the control group proved they were susceptible to the challenge applied. The vaccine PM-3 protected the animals against this challenge and offered a solid immunity and protection after single or double immunization with PM-3. The experience of ours shows a real possibility for successful development of Chlamidial vaccine for sheep. The investigation of the chlamydial genome was of interest to us in connection with choice of suitable vaccinial strains. We compared some of our vaccinial *Chlamydia psittaci* with field chlamydial strains by the use of rep-PCR fingerprinting. Recently, short interspaced repetitive DNA sequences such as repetitive extragenic palindromic (REP) sequences or enterobacterial repetitive intergenic consensus (ERIC) sequences have been shown to be useful in the study of epidemiology (4,5,6,7). REP and ERIC sequences have common characteristics such as highly conserved central inverted repeats and locations in extragenic regions, but differ in sequence size (REP se-

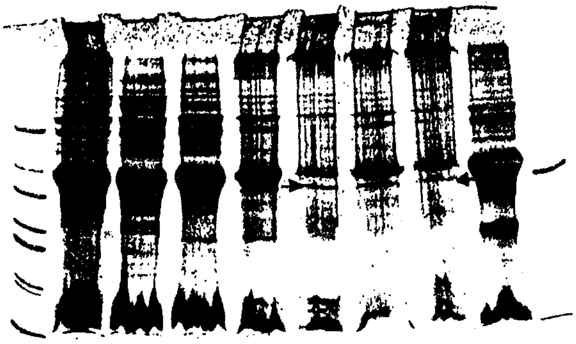

Figure 2

quences-38 nucleotides; ERIC sequences -126 nucleotides) and copy number. These short repetitive sequences have been used for epidemiological studies in a method called repetitive element sequence based PCR (rep-PCR) by Woods et al. (8). In this paper we report the use or rep-PCR to distinguish among 8 *C. psittaci* isolates from sheep (4 of them in the composition of vaccines described) and to compare them with *C. trachomatis-* strains isolated from other animals and 4 *C. trachomatis*-human isolates. The method of amplification and the oligonucleotides used were exactly those described by Verasalovic et al., (9). Figure 3A shows the electrophoresis of rep-PCR amplified products from Chlamydia of different origin. All of the tested "sheep" strains possessed 21 different bands with sizes between 400 and 2100 bp (lines 8-15). Some of the bands were more intense than others. Strains GV (line 13) and P (line 14) were in the composition of vaccines PM-1 and PM-2. They have 4 common bands of 750, 1200, 1600, and 2100 bp. The strains A22 (line 11) and S26/3 (line 12) composed the vaccine strains PM 3. These two strains have 4 sharp common bands, (450, 1200, 1500, and 1600 bp) and 5 common bands between 550 and 800 bp with less sharpness. These four vaccinial strains possessed more or less similar fingerprints with the other *C. psittaci* isolated from sheep (lines 9,10,15,16). They have some common bands with the strains isolated from birds and dog (1.1-8) and *C. trachomatis* (Fig. 3B, 1.8-11) isolated from human (600 bp) as well as *C. psittaci* isolated from pigs (1. 12,13). In all of the tested

Table 2. Bulgarian Vaccine Group Serum Neutralization Assay*

| Ewe No. | Nov 24 | Dec 22 | % Infectivity reduction produced by serum from: Jan 10 | Feb 16 | Mar 1 | Mar 16 | Apr 13 | | Lambed or Aborted | *C. psittaci* positive |
|---|---|---|---|---|---|---|---|---|---|---|
| DPV | 0 | 28 | 56 | 84 | 97 | 112 | 140 | | | |
| DPI | -102 | 174 | -46 | -10 | -5 | 10 | 381 | | | |
| 2080 | 6 | 98 | 100 | 45 | 46 | 70 | 52 | 67 | L | - |
| 2094 | 0 | 33 | 50 | 7 | 12 | 32 | 44 | 73 | L | + |
| 2157 | 0 | 100 | 100 | 1 | 57 | 19 | 23 | 74 | L | + |
| 2172 | 0 | 94 | 100 | 0 | 15 | 68 | 48 | 82 | L | - |
| 2178 | 60 | 96 | 76 | 56 | 57 | 37 | 54 | 99 | L | - |
| 2189 | 0 | 100 | 100 | 32 | 63 | 56 | 29 | 100 | L | - |
| 2220* | 0 | NS | 100 | 0 | 27 | 39 | 25 | 0 | A* | - |
| 2232 | 0 | 100 | 85 | 5 | 51 | 0 | 19 | 83 | L | - |
| 2247 | 0 | 58 | 89 | 3 | 13 | 56 | 58 | 100 | L | - |
| 2259 | 0 | 42 | 97 | 39 | NS | 57 | 67 | 100 | L | - |
| Mean | 6.6 | 80.1 | 89.7 | 18.8 | 37.9 | 43.4 | 41.9 | 77.8 | | |
| (± SD) | 18.9 | 27.6 | 16.2 | 21.7 | 21.0 | 22.3 | 16.7 | 30.1 | | |

*DPV: Days post vaccination; DPI: Days post infection, NS: no sample; L/A-Lambing or Abortion, +: Diagnosis of *C. psittaci.*

Table 3. Treatment: Bulgarian Vaccine

| Ewe # | ELD | L/A | GL | Live | LBD | Dead | % Placenta affected | Chlamydia smear | culture | AFBs in smears vs | pla |
|---|---|---|---|---|---|---|---|---|---|---|---|
| 2080 | 6/5 | L 5/5 | 145 | 1 | 0 | 0 | 0 | - | - | - | - |
| 2094 | 6/5 | L 1/5 | 141 | 1 | 0 | 0 | 50 | + | + | - | - |
| 2157 | 7/5 | L 1/5 | 140 | 1 | 0 | 0 | 0 | + | + | - | - |
| 2172 | 6/5 | L 11/5 | 151 | 1 | 0 | 0 | 0 | - | - | - | - |
| 2178 | 6/5 | L 5/5 | 145 | 1 | 0 | 0 | 0 | - | - | - | ++ |
| 2189 | 7/5 | L 7/5 | 146 | 1 | 0 | 0 | 0 | - | - | - | - |
| 2220* | 6/5 | A 17/4* | 127* | | 0 | | ?* | - | - | - | - |
| 2232 | 6/5 | L 6/5 | 146 | 1 | 0 | 0 | 0 | - | - | - | - |
| 2247 | 6/5 | L 9/5 | 149 | 1 | 0 | 0 | 0 | - | - | - | - |
| 2259 | 9/5 | L 10/5 | 147 | 1 | 0 | 0 | 0 | - | - | - | - |
| 9 | | A = 0 | 145.6 | 9 | 0 | 0 | N grossly | 2 | 2 | 0 | 1 |
| (excl. 2220) | | L = 9 | ±SD 3.47 | | | | affected 1/9 | | | | |
| | | | ±SE 1.16 | | 0 | | x = 6% | 2 | | 1 | |

+: Lambs dying within 2 days of birth due to general weakness; **: Estimated by eye; ELD: Expected lambing date; GL: Actual gestation length; AFBs: Acid fast bacilli (*C. burnetii*) ; L: Lambed; A: Aborted (at lease one lamb born dead or dying within 2 d of general weakness); LBD: live but died; vs: vaginal swab; pla: placenta; *: excluded from analyses due to indication that the cause of abortion was not *C. psittaci;* ?: Placenta grossly abnormal, but proportion affected difficult to assess.

strains we can see different polymorphic bands, typical for every strain. ERIC-PCR resulted a band of 300 bp common for all of the tested strains. This band can be like a marker for Chlamidiae in the diagnostic process. ERIC-PCR is less discriminating than rep-PCR concerning the vaccinial strains. For all the strains tested "ERIC-bands" are about 19. Only 5 of them are sharp enough for comparison. Two of these bands (300 and 850 bp) are possessed by A22 and S26/3 and 5 (400, 500, 700, 850, and 1400 bp) could be seen in GV and P strains. The reproducibility of the method is excellent since the same results were obtained by multiple amplifications of the different DNA preparations. Clus-

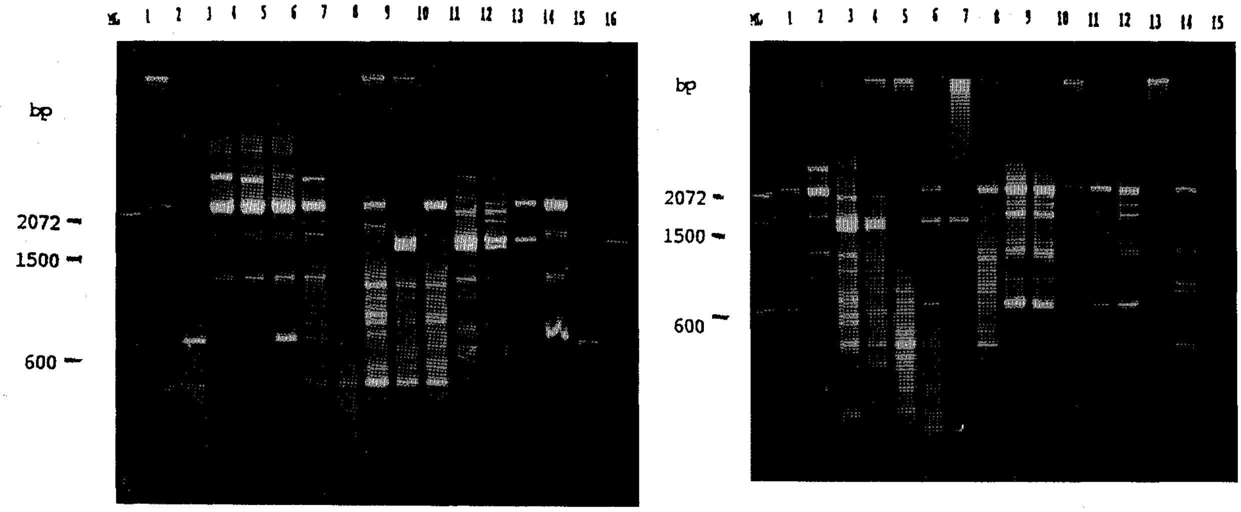

Figure 3

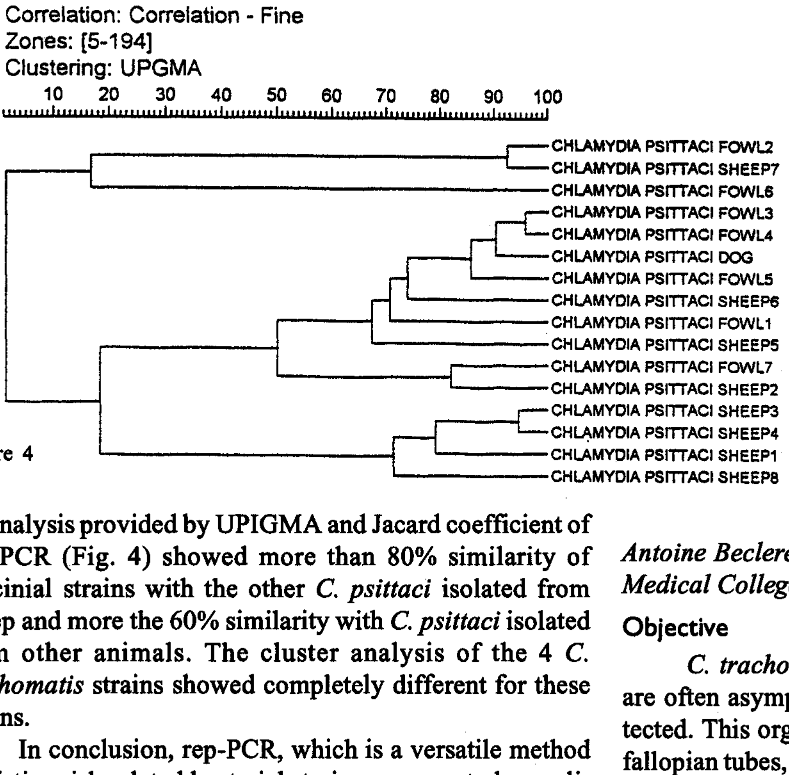

Figure 4

ter analysis provided by UPIGMA and Jacard coefficient of rep-PCR (Fig. 4) showed more than 80% similarity of vaccinial strains with the other *C. psittaci* isolated from sheep and more the 60% similarity with *C. psittaci* isolated from other animals. The cluster analysis of the 4 *C. trachomatis* strains showed completely different for these strains.

In conclusion, rep-PCR, which is a versatile method to distinguish related bacterial strains, appears to be applicable for *C. psittaci.* It is simple and sensitive techniques for the investigation of field and vaccinial strains of *C. psittaci.*

### References

1. Popov G, S Martino. 1983. Vet Sci (Sofia). XX. 1:9-16.
2. Martinov SP, GV Popov. 1992. Proc WAVMI 12th Int. Symposium. Davis. California. Sept. 8-12. 395.
3. Martinov S, G Popov. 1993. Mac Vet Rev. 22 (1-2):25-33.
4. deBruijn FJ, 1992. Appl Environ Microbiol. 58:2180-2187.
5. Geroghiou PR, AM Doggett. et al. 1994. J Clin Microbiol 32:2989-2994.
6. Hulton CSJ, CF Higgins, PM Sharp. 1991. Mol Microbiol 5:825-834.
7. Lows FJ, DW Fulbright et al. 1994. Appl Enviorn Microbiol 60:2286-2295.
8. Woods CR, J Versalovic et al. 1993. J Clin Microbiol 31:1927-1931.
9. Versalovic J, T Koeuth, JR Lupski. 1991. Nucleic Acid Res 19:6823-6831.

### Asymptomatic *Chlamydia trachomatis* Infection and Cervical Cytokines in Women Undergoing In Vitro Fertilization (IVF)

***M Askienazy-Elbhar[1], F Olivennes[2], A Neuer[2], U Lundqvist[2], R Fanchin[2], R Frydmann[2], SS Witkin[3]***

*[1]Laboratoire de Biologie Medicale Magenta, Paris, France, [2] Hospital Antoine Beclere, Clamart, France, [3]Cornell University Medical College, New York, NY USA*

### Objective

*C. trachomatis* infections of the female genital tract are often asymptomatic and, therefore, may remain undetected. This organism is a major cause of occlusion of the fallopian tubes, resulting in infertility or ectopic pregnancy. Many couples with tubal infertility, as well as infertility due to other factors, seek to conceive via IVF. The relation between a past or present chlamydial infection and infertility has remained controversial. Circulating antibodies to *C. trachomatis* have been associated with adverse IVF outcome in some studies, but not in others. A recent study on women undergoing IVF in New York City demonstrated that the presence of cervical IgA antibodies to *C. trachomatis* correlated with poor outcome. To validate and extend these findings, women undergoing IVF in France were tested for circulating and cervical antibodies to *C. trachomatis* and the results correlated with detection of cervical cytokines, clinical findings and IVF outcome.

### Methods

At the time of oocyte retrieval, an endocervical swab was obtained from 120 consecutive women and the contents transferred to a tube containing 0.5 ml sterile saline. Blood was obtained by venipuncture. Serum IgG and IgA antibodies, and cervical IgA antibodies, to *C. trachomatis* were determined by ELISA, using a new commercial kit specific for *C. trachomatis* (Savyon Diagnostics, Beer Sheva, Israel). Interleukin 8 (IL-8), interleukin 10 (IL-10), and interferon gamma (IFNγ) in cervical samples were detected by ELISA.

### Results

Anti- *C. trachomatis* IgA was detected in the cervices of 14 women (11.7%), 45 (37.5%) women had cervical IL8, 15 (12.5%) were positive for IL10 while 54 (45%) had IFNγ. The presence of cervical anti- *C. trachomatis* IgA correlated with detection of IL8 ($p = 0.007$) and IFNγ ($p = 0.04$). IL8 was present in 71.4% of women with and 33.0% of women without anti-chlamydial IgA. Similarly, IFNγ was detected in 71.4% of women with and 41.5% of women without antichlamydial IgA. In contrast, there was no relation between IL10 and cervical anti-chlamydial IgA. The presence of IL8 and IFNγ in the cervix was highly correlated ($p = 0.0001$) and unrelated to the presence of IL10. In marked contrast, there was no relation between circulating anti-chlamydial antibodies and cervical cytokines. In addition, cervical IgA antibodies to *C. trachomatis* were unrelated to the presence of circulating anti- *C. trachomatis* IgG or IgA antibodies. This emphasizes that the cervical IgA antibodies detected were produced locally. As expected, women with occluded or functionally altered fallopian tubes had a higher prevalence of systemic antichlamydial IgG than did women whose infertility was due to other causes ($p = 0.0009$). However, there was no association between cervical anti-chlamydial IgA and cause of infertility. Ongoing pregnancies were documented in 2 (14.3%) women with cervical antichlamydial IgA and in 14 (15.4%) women who lacked this antibody. Similarly, ongoing pregnancies were present in 4 (21%) women with, and in 12 (14%) women without, circulating antichlamydial IgG.

### Conclusions

The association between cervical IgA antibodies to *C. trachomatis* and the presence of IL-8 and IFNγ at this site strongly suggests that measurement of these antibodies identified the presence of an active inflammatory-inducing infection. In the absence of detectable *C. trachomatis* the possibility of a persistent intracellular infection in these women must be considered. In contrast, detection of circulating anti-chlamydial antibodies merely indicated past exposure to this organism. Additional subjects need to be evaluated before the relation between cervical anti-chlamydial IgA and IVF outcome can be evaluated in this population.

---

### Prevalence of *Chlamydia trachomatis* Antibodies in Cord Blood

**[1]S Djukic, [1]M Nedeljkovic, [2]M Pervulov, [3]S Vesic, [2]A Ljubic, [1]B Vranes**

*[1]Institute of Microbiology and Immunology, University of Belgrade, Yugoslavia, [2]Institute of Gynecology and Obstetrics, Clinical Centre of Serbia, Belgrade, Yugoslavia, [3] Institute of Dermatovenereology, Clinical Centre of Serbia, Belgrade, Yugoslavia*

The obligate intracellular bacterium *Chlamydia trachomatis* is an important human pathogen responsible for sexually transmitted diseases throughout the world. This bacterium is a frequent cause of both complicated and uncomplicated genital tract infection (1).

Many chlamydial infections are asymptomatic or not easily recognized on clinical grounds alone (2). The disease process associated with chlamydial infections is thought to be principally immunologically mediated. Repeated episodes of infection clearly play a role in stimulating the host immune response and enhancing damaging pathologic changes (3).

Genital chlamydial infections are an important cause of reproductive morbidity. Various studies have examined the possible roles of maternal *Chlamydia trachomatis* infection in adverse pregnancy outcomes. Suggested complications include premature rupture of membranes, preterm labor and birth, low birth weight (<2500g), intrauterine growth retardation and stillbirth, as well as intrapartum and postpartum maternal and neonatal infectious morbidity (4,5,6).

The aim of this study was to examine 52 fetal blood samples obtained during Cesarean section for the presence of chlamydial IgM and IgG specific antibodies. Pregnant women between the ages of 17 and 41 years (mean 29.14) participated in the study. Prior to Cesarean section mother's peripheral blood was drawn.

The presence of *C. trachomatis* IgM specific antibodies in mother and fetal blood samples was determined by using the indirect immunofluorescence test (Bio-Merieux, France). Tests were set up and performed according to the manufacturer's instructions.

The presence of *C. trachomatis* IgG was determined by using the enzyme-linked immunosorbent assay (Orion Diagnostic, Finland). Tests were set up and performed according to the manufacturer's instructions.

For statistical analysis the Spearman rank correlation were used. A p value $< 0.05$ was considered to be statistically significant.

IgG antibody was detected in 11 of 52 (21.2%) infants. The majority of the IgG-seropositive infants had titers between 1:64 and 1:128. Peak titers of 1:1048and 1:2048 were observed in only 2 of the 52 infants. Nineteen of the 52 (36.5%) women were IgG seropositive. The Spearman rank correlation between IgG titer findings in mother's and fetal blood was significantly high ($r = 0.76$; $p < 0.01$).

Nine of 52 (17.3%) infants were IgM seropositive. All samples had IgM antibody titers of 1:16. IgM antibody was detected in 16 of 52 (30.8 %) women. The Spearman rank correlation between IgM titer findings in mother's and fetal blood was significantly high ($r = 0.74$; $p < 0.01$).

None of the specimens collected in the newborn nursery (< 3 days of care) yielded Chlamydia. These infants had no clinically detectable disease during the first seven days of life.

As it appears that systemic immune function is largely maintained during gestation, maternal susceptibility to infection and the ability to combat an established infection should be similar in both the pregnant and nonpregnant states (7). The circulating B-cell level during gestation is preserved during pregnancy. Numerous studies show normal circulating levels of IgG, IgM, and IgA during uncomplicated pregnancies (8).

Since only IgG is capable of transplacental passage, the presence of chlamydial IgM-specific antibodies in fetal blood show the intrauterine infection (9). It was shown by several authors that whether the infection is symptomatic or asymptomatic, *C. trachomatis*-infected infants regularly develop Chlamydia-specific antibody (10).

Our data indicate that there is a high rate of transmission of *Chlamydia trachomatis* from mother to infant, without any clinical symptoms.

### References

1. K Bergstrom, M Domeika, D Vaitkiene, K Persson, PA Mardh: Prevalence of *Chlamydia trachomatis, Chlamydia psittaci*, and *Chlamydia pneumonia* antibodies in blood donors and attendees of STD clinics. Clin Microbiol Infect 1:253 260, 1996.
2. W Cates, JN Wasserheit: Genital chlamydial infections: Epidemiology and reproductive sequelae. Am J Obstet Gynecol 164:1771 1781, 1991.
3. WL Beatty, RP Morrison, GI Byrne: Persistent Chlamydiae: From cell culture to a paradigm for chlamydial pathogenesis. Microbiol Rev 58:686 689, 1994.
4. JA McGregor, JI French: *Chlamydia trachomatis* infection during pregnancy. Am J Obstst Gynecol 164:1782 1789, 1991.
5. ER Alexander, HR Harrison: Role of *Chlamydia trachomatis* in perinatal infection. Rev Infect Dis 5:713 719, 1983.
6. S Djukic, M Nedeljkovic, M Pervulov, et al.: Intraamniotic *Chlamydia trachomatis* infection. Gynecol Obstet Invest 42:1109 1112, 1996.
7. DF Antezak: Maternal antibody responses in pregnancy. Curr Opin Immunol 1:1135 1140, 1989.
8. BB Feinberg, B Gonik: General precepts of the immunology of pregnancy. Clin Obstet Gynecol 34:3 16, 1991.
9. G Carballal, JB Mahony, C Videla: Chlamydial antibodies in children with lower respiratory disease. Pediatr Infect Dis 11:68 71, 1992.
10. P Francois, P Hirtz, D Rouhan: Transmission materno-infantile de *Chlamydia trachomatis*. Presse Med 18:18 20, 1989.

### Persistence of *Chlamydia trachomatis* and Chlamydial Antigens in HEP-2 and THP-1 Cells after Treatment with Ciprofloxacin

**U Dreses-Werringloer, B Jürgens, H Zeidler, L Köhler**

*Dept. of Rheumatology, Medical School Hannover, Germany*

### Objective

Chronic chlamydial diseases such as Chlamydia-induced arthritis are characterized by persistence of *Chlamydia trachomatis* (CT) in a culture-negative, nucleic acid-positive, metabolically active and viable state at the site of inflammation in mononuclear cells and synovial fibroblasts. Although in one study the duration of early Chlamydia-induced arthritis was shortened by antibiotic treatment (1), other clinical trials using antibiotics even over a prolonged period failed to alter the course of the joint disease (2). Therefore, we started a program to investigate the effect of antibiotics on the low-productivity infection in the monocytic cell line THP-1 in comparison to the replicative-productivity infection in HEp-2 cells. Here, we describe the results with ciprofloxacin, one antibiotic in use for acute and chronic urogenital chlamydial infections.

### Methods

HEp-2 monolayers and 12-O-tetradecanoylphorbol-13-acetate (TPA)-differentiated THP-1 cells were inoculated with *Chlamydia trachomatis* serovar K at a multiplicity of infection (MOI) of 0.05 and 2, respectively. After centrifugation (15 min, 1500 rpm) and incubation for 2 h at 37°C, cells were washed three times and cultured 2 or 3 days in antibiotic-free medium, which contained 0.5 µg/ml cycloheximide for HEp-2 cells. Incubation with ciprofloxacin starting 48 or 72 h post infection (p.i.) was performed continuously or stopped at times indicated. Infected cells were harvested over a culture period of 14-20 days.

### Immunofluorescence microscopy.

Cytocentrifuge smears of cells were stained with a FITC-conjugated murine monoclonal antibody, directed against a common major outer membrane protein (MOMP) epitope of elementary and reticulate bodies. Samples were screened with an epifluorescence microscope and inclusion bodies counted.

### Infectivity of *Chlamydia trachomatis*.

Infectivity was determined by titration of cell lysates on HEp-2 monolayers and subsequent staining of inclusions 48 h p.i. by an immunoperoxidase assay.

### Determination of chlamydial rRNA.

Chlamydial rRNA was measured by an in vitro-hybridization assay with a single stranded chemiluminescence labelled DNA probe.

### SDS-PAGE and immunoblotting

Levels of chlamydial MOMP, lipopolysaccharide

(LPS), and 60-kDa heat shock protein (hsp60), were examined by Western blot analysis. Harvested cells were analyzed by sodium dodecyl sulfate-polyacrylamide gel electrophoresis (SDS-PAGE) (12% or 15% polyacrylamide). Following electrophoresis and transfer to PVDF-membrane, proteins were probed with either anti-MOMP, anti-LPS, or anti-hsp60 antibody.

### Results

The minimal inhibitory concentration (MIC) of ciprofloxacin was determined by simultaneous addition of CT and the antibiotic to cells. The MIC, defined as the lowest concentration of drug which inhibited development of chlamydial inclusion bodies after 48 h, was 0.35 µg/ml for HEp-2 cells and 0.28 µg/ml for THP-1 cells.

Two days after inoculation, chlamydia inclusion bodies had evolved in about 1% of HEp-2 cells. Addition of ciprofloxacin (0.5 µg/ml) 2 or 3 days p.i., to the infected cells led to a continuous decrease of inclusion bodies, infectivity and chlamydial rRNA. Despite the loss of chlamydial infectivity by 10 days p.i., inclusions and rRNA were detectable during further treatment until 20 days after infection. Levels of chlamydial MOMP, LPS, and hsp60 were reduced but still present during the whole culture period. Even treatment with the higher concentration of 1.0 µg/ml starting 48 h after infection did not result in complete eradication of chlamydia from host cells.

Normal growth of chlamydia was obtained when ciprofloxacin (0.5 µg/ml) was omitted 6 or 7 days p.i. The removal of the drug 8 days after infection resulted in a persistent infection, characterized by the maintenance of a constant number of inclusion bodies, and a slight increase in infectivity and levels of chlamydial rRNA, LPS, hsp60, and MOMP.

Inoculation of TPA-differentiated THP-1 cells with CT resulted in a low-productivity infection. In about 0.3-0.5% of the cells inclusion bodies were demonstrated 48 h after infection. Treatment with ciprofloxacin (0.3 µg/ml) starting 2 or 3 days postinfection significantly diminished the number of inclusions and chlamydial infectivity. After 9 or 10 days (= 12 days p.i.), according to the time ciprofloxacin had been added, no typical inclusions were seen. Loss of chlamydial infectivity had already occurred 7 or 8 days p.i. Although no chlamydial inclusions and infectivity were present, single chlamydial particles, LPS, and hsp60 were detected over the whole culture period of 14 days. Even exposure to a higher concentration (0.5 µg/ml) did not lead to complete eradication of chlamydia.

### Conclusions

Ciprofloxacin fails to eradicate chlamydia completely from HEp-2 and THP-1 cells. Although chlamydial growth is inhibited, inclusions or particles and the antigens LPS, hsp60, and MOMP persist over a period of 20 days in HEP-2 and 14 days in THP 1 cells. Failure of complete eradication does not appear to be an effect of a too low concentration of antibiotic.

Productive infection in HEp-2 cells is changed to a persistent infection by short-term treatment with ciprofloxacin.

The in vitro persistence of chlamydial LPS, hsp60, MOMP, and chlamydial particles in THP-1 and HEp-2 cells may be of pathogenetic relevance for in vivo induction and perpetuation of chronic chlamydial diseases. Further studies are under way to elaborate the ability of other antibiotics (azithromycin, doxycyclin) to eradicate CT in vitro.

### References

1. Lauhio A, Leirisalo-Repo M, Lähdevirta J, Saikku P, Repo H: Double-blind, placebo-controlled study of three-month treatment with lymecycline in reactive arthritis, with special reference to *Chlamydia trachomatis*. Arthritis Rheum 1991; 34:6 14.
2. Toivanen A, Yli-Kerttula T, Luukkainen R., Merilahti-Palo R, Granfors K, Seppälä J: Effect of antimicrobial treatment on chronic reactive arthritis. Clin Exp Rheumatol; 11:301 397.

### Failure to Detect *C. trachomatis* by MOMP PCR, in Contrast to Cultures with Multiple Passages, In Situ Hybridization and Immunoperoxidase Staining, In Pelvic Samples of Women with Tubal Factor Infertility

***J Henry-Suchet [1], M Askienazy [2], J Orfila [3], M Thomas [3], Jf Lefebvre [3], F Eb [3], Dl Patton [4], La Campbell [4]***

*[1] Hospital Jean Rostand - Sevres - France - [2] Magenta Laboratory - Paris - France - [3] Microbiologic Laboratory University Of Amiens - France - [4] University Of Washington - Seattle - Washington - USA*

*C. trachomatis* detection from pelvic samples in chronic infection and more particularly tubal factor infertility (TFI) cases is controversial, chlamydial persistence being supposed to maintain the host immune response.

We obtained, in previously published assays, 30% positive cultures, and were able to keep 7 isolates, from 18/58 pelvic samples taken by laparoscopy in 7/21 TFI cases with positive CT serology. According to the sampled site, we obtained positive culture in 7/13 (53%) tubal biopsies, 8/21 (38%) adhesion biopsies, 3/13 (23%) tubal smear and 3/17 (17%) peritoneal cul-de-sac fluid (1). Of this series, 11 samples from 3 culture positive cases, were tested by MOMP PCR, only 3/11 were found positive. In contrast, the isolated strains were found positive, when tested in MOMP PCR.

In a new series of 146 pelvic samples taken in 55 TFI cases, all attempts to detect CT by PCR and culture were negative (2), similar to other authors findings.

Using in situ detection, the presence of chlamydial DNA in pelvic samples was detected in 1/10 cases by electron microscopy, 3/29 (10%) by culture, 13/23 (50%) by in situ hybridization (ISH) and 16/26 (61.5%) by immunoperoxidase staining (IP) (3).

### Objectives

To compare chlamydial agent detection by MOMP PCR and other methods.

### Material and methods

Nine tubal biopsies taken on 9 pathological tubes, were studied as follows: 1) cell culture with 7 successive passages; 2) MOMP PCR; 3) ISH based on a *C. trachomatis* 7.4 kb plasmid cloned into Ptz 18r digested with Eco R1 and a Ptz 18r digested with Eco R1 as negative control probe; and 4) IP staining on 4 μm tissue sections, incubated in unconjugated Chlamydia monoclonal antibody (mAB) KK-12 at various dilutions. This mAB recognizes a species specific determinant on the major outer membrane protein of *C. trachomatis*.

### Results

In this short series, chlamydia detection was positive in 1/9 cases by ISH, 7/9 cases by IP and in contrast, no case was positive by PCR nor by culture.

### Comments

This assay shows that persistent chlamydial agent in chronic pelvic female infections is difficult to cultivate and is not recognized by MOMP PCR. In addition, pelvic samples probably have PCR inhibitors. Samples were not processed the same way for PCR and ISH/IP (tissue processing). Other amplification methods, based on different epitopes should be of interest in further studies.

### References

1. Askienazy-Elbhar M. Moncan T. Orfila J. Henry-Suchet J. Tannous W. Gautheron C. Cultures de prélèvements pelviens dans des chlamydioses aiguës et chroniques. Etude des sérotypes obtenus. ISSTDR proceed. Banff Canada , 1991
2. Thomas M. Apport de la biologie moléculaire à l'épidémiologie et à la physiopathologie de l'infection chlamydienne. Thèse UTC Compiègne , 1/320 1996
3. Patton D.L. Askienazy M. Henry-Suchet J. et al. Detection of *Chlamydia trachomatis* in fallopian tube tissue in women with postinfectious tubal infertility. Am J Obstet Gynecol 171:1, 95-101 1994

---

### Variability of Non-Culturable *Chlamydia trachomatis* Persisting in Human Peripheral Blood Monocytes and Human Monocytic THP-1 Cells

***L Koehler[1], E Nettelbreker[1], A Hudson[2], H Gérard[2], D Bonk[1], H Zeidler[2]***

*[1]Medical School Hannover, Hannover Germany, [2]Medical College of Pannsylvania, Philadelphia, USA*

### Introduction

After urogenital infection with *Chlamydia trachomatis* serovar D-K 1–3% of the patients develop a reactive arthritis. Diagnosis of chlamydia-induced arthritis is based in part upon detection of various chlamydial macromolecules in synovial fluid and/or synovial membrane, although the organism can not be grown by standard microbiological techniques. Intracellular chlamydia have been demonstrated within monocytes isolated from synovial fluid and membrane in a culture-negative, nucleic acid positive-state, even as late as 12 years after onset of the disease. Culture-negative, nucleic acid-positive chlamydial infections of the joint have been designated "inapparent" to suggest that the organism persists at this site in an intact, possibly viable form. It has been proposed that the persistence of *Chlamydia trachomatis* in monocytes is relevant to the pathogenesis of inflammation in chronic chlamydial diseases. Previously, we have shown that *Chlamydia trachomatis* persists in a non-replicative state in human peripheral blood monocytes and gamma interferon (IFNγ) treated human monocytic THP-1 cells.

### Methods

Human peripheral blood monocytes obtained from healthy donors were prepared by Ficoll gradient centrifugation and vigorous washing. Human monocytic THP-1 cells were differentiated by addition of the phorbol ester TPA at a concentration of $2 \times 10^{8}$ M. *Chlamydia trachomatis* serovar K was added to the cells at the multiplicity of infection (MOI) of 1. THP-1 cells were incubated with or without IFNγ 24 h prior to infection. *Chlamydia trachomatis* -infected cells were harvested at different time points.

### Immunofluorescence microscopy

Cytocentrifuge smears of the cells were prepared and incubated with a fluorescein-conjugated murine monoclonal antibody, directed against a common major outer membrane protein (MOMP) epitope of elementary and reticulate bodies. The samples were screened with an epifluorescence microscope. Number of inclusion bodies were counted and expressed as inclusion bodies/$10^{5}$ cells.

### Infectivity of *Chlamydia trachomatis*

Infectivity of *Chlamydia trachomatis* was determined by titration of the lysates of infected human monocytes and THP-1 cells on Hep-2 cells using an indirect immunoperoxydase assay (IPA). The number of inclusion bodies/volume was expressed as inclusion forming units per ml (OFU/ml).

### Molecular analyses of the chlamydial 16s rRNA gene

DNA and RNA were prepared from cell pellets, and subjected to standard (screening) PCR or RT-PCR. For RT-PCR reactions, 1 μg RNA was reverse transcribed using the MuLV enzyme and the downstream primer. The downstream primer used in all analyses was 30-mer corresponding to bases +333 to +363 (5'CTGCAGCCTCCGTAGAGGTCTGGGCAGTGTC-3') . The upstream primer used for standard PCR corresponds to bases -103 TO -73 (5'-AAGCTTTTCTTAACAATGCAAATGAGATAG-3') and for the RT-PCR to nucleotides -66 TO -44 (5'-GCCAGTATAGATGCTTGTGAGGA-3') of the chlamydial rRNA. Both upstream primers lie in a non-coding region unconserved in sequence except among serovars of *Chlamydia trachomatis*. Products were analyzed on agarose gels, and visualization of products was via ethidium bromide staining; positive/negative controls were included in each assay.

### Results

Infection of human blood monocytes with *Chlamydia trachomatis* was non-replicative as shown by titration of the lysates of the infected monocytes on Hep-2 cells but chromosomal DNA of *Chlamydia trachomatis* was present during the whole culture period of 10 days when standard PCR was performed on DNA preparations from infected monocytes. To assess the viability of the intracellular chlamydia, RT-PCR analyses were performed specific for primary rRNA transcripts which are only produced by metabolically active organisms. The presence of these primary rRNA transcripts was demonstrated in monocytes from day 3 to day 10 post infection. Preparation of peripheral blood monocytes does not exclude contamination with other blood cells such as lymphocytes which are a known producer of IFNγ. IFNγ has been shown to induce persistent chlamydial infection in human epithelial cells and fibroblasts via tryptophan degradation (3). Therefore, the human cell line THP-1 was used as a model for pure monocytic cells. In contrast to human blood monocytes, inoculation of TPA-differentiated cells with *Chlamydia trachomatis* resulted in low-productivity infection. During the culture period of 10 days inclusion bodies were observed in 1–2% of the THP-1 cells. Electron microscopic analyses revealed the presence of single morphological atypical chlamydia resembling those observed in human blood monocytes. By preincubation with IFNγ the number of inclusion bodies was reduced by 90% and the replicative infection was converted into a non-replicative one. The number of inclusion bodies and the infectivity was suppressed by IFNγ in a dose dependent manner. Chlamydial growth was completely inhibited at a concentration of 25U/ml IFNγ. To prove the presence of viable chlamydia after IFNγ treatment within the THP-1 cells IFNγ was withdrawn at various times after infection, resulting in a rise of the number of inclusion bodies and of the infectivity.

### Conclusions

So far viability of *Chlamydia trachomatis* has been defined by detection of infectious organism, lack of infectivity has been understood to mean non-viability. Our data show that chlamydia infecting human blood monocytes are viable within the host cell, even though the infection is non-replicative and arrested at some point in their life cycle. A similar state was induced by IFNγ in chlamydia-infected THP-1 cells. However, morphological atypical chlamydia were also found in untreated infected THP-1 cells suggesting that persistence of *Chlamydia trachomatis* is mediated via IFNγ-dependent and -independent mechanisms.

The presence of metabolically active chlamydia in monocytes may contribute to the pathogenesis of chronic chlamydial diseases via delivery of synthesized chlamydial antigens inducing and/or sustaining the inflammatory response.

### References

1. Schmitz E, Nettelbreker E, Zeidler H, Hammer M, Manor E, Wollenhaupt J. Intracellular persistence of chlamydial major outer membrane protein, lipopolysaccharide and rRNA after non-productive infection of human monocytes with *Chlamydia trachomatis* serovar K. J Med Microbiol 38:278–285, 1993.
2. Dreses-Werringloer U, Bonk C, Reiss G, Zeidler H, Koehler L Effect of ciprofloxacin and interferon-γ on the infection of *Chlamydia trachomatis* in the monocytic cell line THP-1. Br J Rheumatol 35(suppl 1):169, 1996.
3. Beatty WL, Belanger RA, Desal AA, Morrison RP, Byrne GI. Tryptophan depletion as a mechanism of gamma interferon-mediated chlamydial persistence. Infect Immunol 64:3705–3711, 1994.

---

### Human Heat Shock Proteins In First Trimester Human Decidua

***A Neuer[1,3], P Ruck[2], K Marzusch l, J Dietl[1], E Kaiserling[2], H-P Horny[2], S.S Witkin[3]***

*[1]Dept of OB/GYN University of Tübingen, Germany,*
*[2]Institute of Pathology University of Tübingen, Germany,*
*[3] Dept of OB/GYN, Div.of Immunology and Infectious Dis.,Cornell University Medical College New York, NY.*

Introduction: Heat shock proteins (hsp) are induced in cells after exposure to stressful stimuli, such as a temperature increase or infection. During normal cell growth these proteins are involved in essential cell functions such as facilitating protein translocation across membranes, protein folding and binding to hormone receptors. Hsp have been highly conserved during evolution, leading to structural and sequence homology between bacterial and human proteins in the same family. The adverse reproductive sequelae of tubal infertility and ectopic pregnancy due to *C. trachomatis* infection have been associated specifically with an antibody response to the 60 kD chlamydial hsp. It has also been shown, that the pregnancy rate after IVF in women previously sensitized to the chlamydial hsp is markedly reduced (1). Hsp have been classified according to their molecular mass, as determined by sodium dodecyl sulphate-polyacrylamide gel electrophoresis (SDS-PAGE) rather than by function. During acute and latent chlamydial infections a variety of hsp are expressed by Chlamydia, leading in susceptible patients to the development of autoimmune phenomena (e.g. implantation failure). The aim of this study was to immunolocalize possible binding sites for crossreacting anti-hsp antibodies in the decidua of normal early human pregnancy. Therefore the distribution of the 4 most common human hsp was analysed by immunohistochemistry.

### Materials and Methods

Decidual tissue from 5 healthy normal pregnant women undergoing elective termination of pregnancy at 7-11 weeks' gestation was investigated. No evidence of infection was found by clinical examination, bacterial cultures and cervical Chlamydia DNA-probe testing. Fresh frozen decidual tissue sections were immunostained by the avidin-biotin-peroxidase complex (ABC) method with monoclonal antibodies against human hsp 27, hsp 60, hsp 72 and hsp 90 (Stressgen, Victoria B.C.). 3,3' diaminobenzidene (Sigma, Munich, Germany) was used as a chromogen. For negative control the primary antibody was replaced with Tris-buffered saline and normal mouse serum.

Table 1. Distribution of heat shock proteins in human decidua

| Cell type | HSP27 | HSP 60 | HSP 70 | HSP 90 |
|---|---|---|---|---|
| Stromal cell | +-+++ (5/5) | +/+ (3/5) | +-+++ (5/5) | +/- |
| Endometrial glands | + (2/5) | +-++ (2/5) | ++/+++ (5/5) | +/- |
| Leukocytes | - | - (5/5) | +-+++ | +/- |

Numbers in parenthesis refer to the number of positive samples / total number of samples examined

### Results

Decidual stromal cells stained for hsp 27 and hsp 72 in all cases, whereas staining for hsp 60 was seen in 3 cases. The epithelium of endometrial glands exhibited immunoreactivity for hsp 27 and hsp 60 in 2 and for hsp 72 in all cases. Leukocytes were immunoreactive for hsp 72. Smooth muscle cells in the wall of blood vessels stained for hsp 60 in 3, and for hsp 72 in all cases. No unequivocal staining for hsp 90 was seen. A summary of the results is illustrated in table 1.

### Discussion

Heat shock proteins are among the first major products of zygotic gene activity in mouse embryogenesis, corresponding to a time of rapid development (2) Little is known about the physiology of hsp expression in human embryo development and early pregnancy. Controversy surrounds the possible role of hsp in autoimmune disease, because of the immunological similarity between microbial and human hsp. It has been suggested that hsp may provide a link between bacterial infection and the development of autoimmune phenomena, resulting in infertility and implantation failure (3). Here we present preliminary data, demonstrating further evidence for a differentiated expression of hsp in early pregnancy. Hsp 27, which has been implicated as a regulator of actin cytoskeleton organization was found mainly expressed in decidual stromal cells. This suggests that the regulation of the hsp 27 expression is different in decidual epithelial and stromal cells. Hormonal factors, especially the elevated progesterone levels in pregnancy, may be important in this respect. Studies looking at the distribution of hsp27 in endometrium of premenopausal women at different stages of their menstrual cycle and endometrium from postmenopausal women receiving hormone replacement, indicated that hsp 27 is increased by estrogen and inhibited by progesterone in glandular epithelium but not stroma. (4) The intense staining and widespread distribution of hsp 60 and hsp 70 observed in our study is consistent with current knowledge regarding the essential role of these proteins in protein folding and translocation between intracellular compartments. This result also supports data by Tabibzadeh et al. who recently showed the existence of the full complement of hsp in endometrium of healthy women (5). In accordance with our observations a similar hsp distribution profile and the dependency of hsp expression on endocrine parameters of the menstruation cyclus was described. Hsp 90 which has been related to interactions with protein substrates, including steroid receptors was found nonspecifically distributed both in stromal and epithelial cells. Whether this variable staining pattern is due to fluctuations in the systemic endocrine milieu, inconsistencies in the applied technique, or to the impact of other factors remains to be determined. However, growing evidence exists that chronic and/or repeated bacterial infections and excessive exposure to bacterial (e.g. chlamydial) hsp can trigger immunity to hsp in the genital tract. In this regard the crossreactivity between human and mycobacterial and chlamydial hsp 60 has been most established. Previous sensitization to the chlamydial hsp combined with relative overproduction of human hsp during the early stages of pregnancy may lead to reactivation of hsp-sensitized lymphocytes and the subsequent induction of an inflammatory response. This will disturb immune regulatory mechanisms necessary for embryo implantation or survival and may lead to immune rejection. Alternatively decidual cell populations expressing human hsp can become the target of a humoral immune response. The human decidua may therefore serve as target tissue for postulated crossreacting anti-hsp antibodies.

### Acknowledgement

The outstanding intellectual support, technical expertise and critical review by Jan Jeremias is gratefully acknowledged. Supported in part by grant Deutsche Forschungsgemeinschaft Ne 602/ 1-1

### References:

1. Witkin S.S., Sultan K.M., Neal G.S., Jeremias J., Grifo J.A., Rosenwaks Z.: Unsuspected *Chlamydia trachomatis* infection and in vitro fertilization outcome. Am J Obstet Gynecol. 1994, 171; 1208-1214.
2. Bensuade O, Morange M: Spontaneous high expression of heat-shock proteins in mouse embryonal cells and ectoderm from day 8 mouse embryo. EMBO J 2:173-177, 1983.
3. Witkin S.S., Jeremias J., Neuer A., David S., Kligman I., Toth M., Willner E., Witkin K.:Immune recognition of the 60kD heat shock protein: Implications for subsequent infertility. Inf Dis Obstet Gynecol 4:186, 1996.
4. Padwick ML; Whitehead M; King RJ: Hormonal regulation of HSP27 expression in human endometrial epithelial and stromal cells. Mol Cell Endocrinol 1994, 2; 9-14.
5. Tabibzadeh S., Kong Q. F., Satyaswaroop P.G., Bakaknia A.: Heat shock proteins in human endometrium throughout the menstrual cycle. Hum Reprod 1996, 11: 633-640.

### Is There a Connection Between Immunity Response of Women With *Chlamydia trachomatis* Infections of the Genital Tract and Serum or Cervical Mucus Antiseminal Antibodies?

**T Miroshnikova, L Chernyshova,**
*Donetsk State Regional Centre of Maternity and Child Protection, Donetsk, Ukraine*

### Introduction

Frequently immune reactions to different kinds of invasion is the cause of aggression against gametes, their physiological interaction and movement or against the embryo. As known, infection of the genital tract is one factor leading to sensitization to sperm. Infectious agents may play the role of trigger. Some of them have antigenic properties which are similar to those of spermatozo, this occurs most frequently in STDs.

### Objective

To establish the connection between the cell immune response of women with *Chlamydia trachomatis* infections of the genital react and serum or cervical mucus antiseminal antibodies.

### Methods

Immunological status were determined by immunofluorescence with monoclonal antibodies to $CD3^+$(common T-lymphocytes), $CD4^+$ (T-helper/inductors), $CD8^+$ (T-suppressor/cytotoxic cells), $3F3^+$ (B-lymphocytes) immune cells. Blood samples were obtained by venipuncture from 105 women on the 25–27 day of the menstrual cycle. Identification of *Chlamydia trachomatis* was performed by immunofluorescence with monoclonal antibodies. Antiseminal antibodies were determined in women's serum and cervical mucus by Friberg test, Shuvarsky test, Kuretsrok-Muller test.

### Results

32.2% of the women had *Chlamydia trachomatis* infection of the genital tract during 1 year; 56.6% of the women had this infection during 2–4 years and 35.6% during 5–7 years.

Persistent *C. trachomatis* in the genital tract during 5–7 years led to an increase in the relative quantity of CD4+ by 7.45%, and a decrease of CD8+ from 29.0% to 21.77%. The measurement CD4:CD8 was increased by 54.5%. The relative quantity of B-lymphocytes didn't depend on inflammatory duration.

Women with *C. trachomatis* infection of the genital tract during 5–7 years were divided into two groups. Serum and cervical mucus antiseminal antibodies were demonstrated in 12 (33%) women with *C. trachomatis* genital infection. They were described as being Group I. In Group II there were 15 patients without antiseminal antibodies.

The inquiry of cell immunity in these two groups and that of healthy women is demonstrated in Table 1.

The content of CD3+ cells in patients with *C. trachomatis* and antiseminal antibodies (Group I) was lower by 9.6% compared with those of women without antiseminal antibodies (Group II), and by 10% in those of healthy women ($P<0.05$). The contents of CD4+, CD8+ cells in Group I were the same as they were in the other groups ($P>0.05$). The measurement of CD4:CD8 and relative quantity of 3F3+ cells in women with *C. trachomatis* infection of the genital tract and antiseminal antibodies were highest among these three groups of women ($P<0.05$).

### Discussion

Recently scientific studies concerning the influence of *C. trachomatis* on cell immunity have been published. In some of them it was shown that genital infection increased the immune response to seminal antigens (Chernyshov VP., et al, 1990; 1993).

According to data received in our laboratory, persistent *C. trachomatis* infection of the genital tract in women over a long period (5–7 years), leads to changes the cell immunity in some and leads to an increase in the relative quantity of T- and B-lymphocytes, and the measurement of CD4:CD8. These immunological changes predispose patients to the development of antiseminal immunity.

### Conclusion

Persistent *C. trachomatis* infection in the female genital tract with a duration of 5–7 years leads to a change in cell immune responses which may cause the appearance of antiseminal antibodies.

Table 1. The content of CD3+, CD4+, CD8+, 3F3+ cells and measurement of CD4:CD8 of healthy women and patients with genital Chlamydia trachomatis infection.

| N | Group | CD3+ cells | CD4+ cells | CD8+ cells | CD4:CD8 | 3F3+ cells |
|---|---|---|---|---|---|---|
| 1 | Group I (n=12) | 52.3±5.4 | 26.7±4.2 | 18.2±5.4 | 2.4±0.2 | 24.0±2.1 |
| 2 | Group II (n=15) | 61.9±3.5 | 32.3±5.5 | 23.7±4.2 | 1.4±0.4 | 15.8±3.1 |
| 3 | Healthy women (n=17) | 63.2±4.2 | 30.2±5.1 | 20.8±4.8 | 1.2±0.3 | 12.2±2.8 |
| | P1–2 | <0.05 | >0.05 | >0.05 | <0.05 | <0.05 |
| | P1–3 | <0.05 | >0.05 | >0.05 | <0.05 | <0.05 |
| | P2–3 | >0.05 | >0.05 | >0.05 | >0.05 | >0.05 |

### Habitual Abortion – The Consequence of Immune Response to *Chlamydia trachomatis* Infection of the Upper Genital Tract in Women

**T Miroshnikova**

*Donetsk State Regional Centre of the Maternity and Child Protection, Donetsk, Ukraine*

### Introduction

The interest of studying the effect of chlamydial infection on the duration of pregnancy and deliveries is such because *Chlamydia trachomatis* as pathogenic agent is the cause of 40–60% of infections of the upper genital tract. *Chlamydia trachomatis* infects women during their reproductive age and a period of intense sexual activity and leads to prematurity and preterm deliveries. Under this condition, chronic activation of the immune system in women with *Chlamydia trachomatis* upper genital tract infection may become a major mechanism of habitual abortion.

### Objectives

To establish the role of immune response to *Chlamydia trachomatis* upper genital tract infection in women with habitual abortion.

### Methods

Immunological status was determined by immunofluorescence with monoclonal antibodies to CD3+ (common T-lymphocytes), CD4+ (T-helpers/inductors), CD8+ (T-suppressors/cytotoxic cells), 3F3+ (B-lymphocytes) immune cells and by absorbable reaction with polyethelenglycole-6000 in order to evaluate the maintenance of immune complexes (IC). Blood samples were obtained by venipuncture of 48 pregnant women with habitual abortion and chronic genital chlamydiosis and 22 healthy ones in the 1st trimester.

### Results

Two groups were compared by age, marital status and quantity of sexual partners. 19.2% pregnant women with *C. trachomatis* upper genital tract infection had 1 abortion before this pregnancy, 25.4% patients had 2 abortions and 25.4% women had 3 or more. Duration of pelvic inflammatory disease was 1 year in 32.2% of women, during 2–4 years in 34.2% of patients and during 5–7 years in 35.6%.

According to our data, the physiologic course of pregnancy in Ist trimester was characterized by reduction of absolute quantity of CD3+, CD4+, and CD8+ cells and increase of relative level of B-lymphocytes.

This condition of immunosuppression was stable in pregnant women ≥ 24 years old. In pregnant women age ≤ 20 years, relative contents of CD3+, CD4+, and CD8+ cells were higher than those of nonpregnant women the same age.

Pregnant women with *C. trachomatis* upper genital tract infections had higher absolute (by 2 times) and relative quantity (19.6%) of B-cells and relative quantity of CD3+ (12.61%). The measurement of CD4/CD8 of women with genital Chlamydiosis was increased by 1.2 times over those of healthy pregnant subjects.

In pregnant women with chronic Chlamydiosis the relative contents of CD3+, CD4+, CD8+ lymphocytes increased directly proportionally to age and did not depend on the duration of the inflammatory process.

Relative level of CD3+, CD4+, CD8+ immunoregulator cells in pregnant subjects < 26 years of age, with *C. trachomatis* upper genital tract infection was higher than those of non pregnant subjects on the average of 25.5% (CD3+), 9.7% (CD4+), 12.6% (CD8+), and higher than in healthy pregnant subjects by 15.2% (CD3+), 7.7% (CD4+), 8.2% (CD8+).

Concentration of IC in blood serum of pregnant women with *C. trachomatis* upper genital tract infection was 40.93 condition units (cond.un.). It was higher than healthy pregnant subjects on the average of 48.3% cond.un.

The level of IC in 16 year old pregnant subjects with C trachomatis upper genital tract infection was 39.0 cond. un., and was 47.3 cond. un with patients of 36 years of age.

The content of IC in blood serum of pregnant women with inflammatory process during 2–4 years was 38.5 cond. un., and was 44.2 cond. un. if chronic Chlamydial infections persisted in upper genital tract during 5–7 years.

### Discussion

The unusual changes revealed in T-cell subpopulations of lymphocytes in women suffering from prematurity with a background of genital Chlamydiosis during pregnancy has, inour opinion a double explanation.

First, populations of CD8+ cells are heterogeneous. It includes besides T-lymphocytes with suppressor activity, cytotoxic cells and EK-cells. It is rather difficult to separate functionally these populations (Barishnikov A.Y., et al., 1989).

Cytotoxic cell [effector lymphocytes (T-killers) or sensitized lymphocytes in old nomenclature] are able to destroy target cells in the absence of specific antibodies and complement.

As follows, T-killers influence allogeneic cells of donors against which they are sensitized by previous introduction of cells or tissue. As far as the interaction of T-lymphocytes-killers with target cells containing appropriate antigens is being executed by direct contact, this mechanism is one of the basic reactions of transplant rejection–that is the fetus (Vershigora A.E., 1990).

Thus, increase contents of CD8+ lymphocytes with women with *C. trachomatis* infection of upper genital tract in Ist trimester of pregnancy is probably due to growth of cytotoxic cells sensitized to the tissues of the fetus as an allotransplant which is clinically expressed by the threat of pregnancy interruption or self abortion.

On the other hand, such high level of CD8+ lymphocytes in pregnant women suffering from habitual abortions with a background of chronic genital Chlamydiosis may be explained by compensative changes in the immune system in response to insufficient functional activity of T-suppressors (CD8+ cells) which is confirmed by twice higher contents of relative and absolute quantity of B-lymphocytes-antibodies of producing cells at constant level of T-helpers/ inductors (CD4+).

### Conclusion

Chronic *C. trachomatis* upper genital infections in pregnant women changes their immunological reactions and may be the cause of habitual abortion.

### SeroCT™ – A New Peptide Based ELISA Test for Specific Detection of *Chlamydia trachomatis* Antibodies

**B Ohana, V Prankratova*, A Groh*, E Straube*, R Brand, A Blaicher, I Mezamer-Tov, O Horovitz, and M Lipson**

*Department of Research and Development, Savyon Diagnostics, Kiryat Minrav, Ashdod, 7701, Israel, *Institute of Medical Microbiology, Friedrich Schiller University of Jena, Germany*

Serological crossreactions frequently occur between the different species of Chlamydia such as *C. trachomatis* and *C. pneumoniae*. Since a large proportion of the population has been exposed to *C. pneumoniae*, the prevalence of anti-Chlamydia antibodies is high and the differentiation between *C. pneumoniae* and *C. trachomatis* specific antibodies using conventional screening tests is not accurate. To improve the diagnostic value of *C. trachomatis* serology, SeroCT a *C. trachomatis* species-specific peptides-based ELISA assay has been developed. All the peptides which are used as antigen in SeroCT, originated from major immunogenic proteins, have no homology with C. pneumonaie yet, share sequence homology with all the 15 *C. trachomatis* serovars.

Table 1. The prevalence of *C. trachomatis* antibodies as was determined by SeroCT and MIF on different patients' groups

| Group & no. patients | SeroCT+ | | MIF+ | | Overall agreement[a] | |
|---|---|---|---|---|---|---|
| | IgG | IgA | IgG | IgA | IgG | IgA |
| Arthritis (46) | 53 | 34 | 56 | 46 | 88 | 82 |
| Cervicitis+ (53) | 82 | 26 | 87 | 53 | 100 | 91 |
| Tubal infertility (36) | 92 | 42 | 97 | 67 | 100 | 87 |
| Adnexitis+ (i21) | 100 | 48 | 100 | 52 | 100 | 86 |

+ Culture positive

[a] Overall agreement was calculated according to the agreement between SeroCT and MIF FOR both positive and negative sera, while sera that were positive on both *C. trachomatis* and *C. pneumoniae* according to MIF (inconclusive interpretation of results) were excluded.

SeroCT is highly specific as compared to different MIF assays, see Figure 1. Specificity of above 90% was

Fig 1: Specificity of SeroCT as compared to 3 different MIF assays

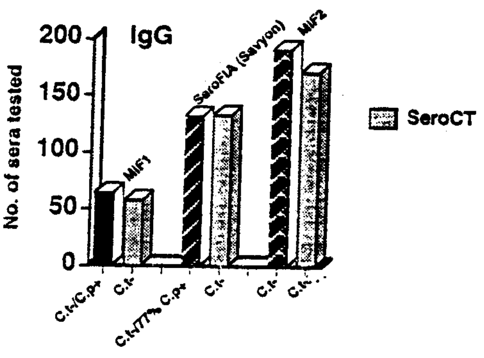

MIF1&2 are in house MIF references performed by 2 independent laboratories. SeroFIA, a new microimmunofluorescence assay for the differential detection of C. trachomatis, C. pneumoniae, and C. psittaci. Different groups of sera were screened by each MIF assay.

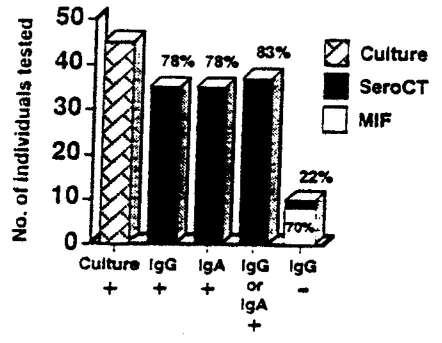

Fig 2: Sensitivity of SeroCT as compared to culture observed on sera with high prevalence of *C. pneumoniae* antibodies according to MIF. The sensitivity of SeroCT on a culture-positive population preselected by MIF, was 78% for both IgG and IgA, see Figure 2. Out of the 22% sera that were negative by SeroCT-IgG, 70% were also negative by MIF. Moreover, on clinically defined populations (pelvid inflammatory disease, cervicitis, arthritis, and tubal infertility), high prevalence of IgG *C. trachomatis* antibodies was observed in both SeroCT and MIF, as was expected. A lower prevalence of IgA *C. trachomatis* antibodies was observed in SeroCT as compared to MIF, probably due to the residual cross reactivity of the antigen that is used in MIF. A high overall agreement between SeroCT and MIF was observed for both IgG (88%–100%) and IgA (82%–91%), (Table 1).

The high specificity and sensitivity of the SeroCT for the specific detection of *C. trachomatis* antibodies makes this new generation test, an accurate, efficient and cost effective screening tool for the diagnosis of *C. trachomatis* and its distinction from *C. pneumoniae* infections.

### Serum Antibodies to a Conserved B-Cell Epitope of Chlamydial Heat Shock Protein 60 (hsp60) in Patients with Sexually Acquired Reactive Arthritis (SARA)

**K Domeika[1], SS Witkin[2], P-A Märdh[1], and M Domeika[1]**

[1]*Institute of Clinical Bacteriology, Uppsala, Sweden,* [2]*Department of Obstetrics and Gynecology, Cornell University Medical College, New York, USA*

### Objectives

Hsp60 is phylogenetically conserved and exhibits a high degree of sequence homology between eucaryotic cells, bacteria and within th C. trachomatis species (1). Antibodies to chlamydial hsp60 have been demonstrated in C. trachomatis eye, respiratory- and genital tract infections (2), as well as in patients with reactive arthritis having a history of primary C. trachomatis infection (3). Stimulation with a foreign antigen may elicit cross-reactivity with self-antigens, (4) and lead to the local accumulation of heat shock or stress proteins. This may provide the trigger for immunopathologic processes such as arthritis, tissue damage in salpingitis and scarring in trachoma. Our study was aimed at investigating the immune response to two synthetic peptides corresponding to sequences of the chlamydial hsp60 that are unique to this organism (amino acids 151-162) and conserved sequences that are also expressed in the human hsp60 (amino acids 260-271), in patients with SARA.

### Materials and Methods

Thiry-nine samples from C. trachomatis microimmunofluorescence (MIF) IgG antibody-positive patients with diagnosed SARA were selected from the previous study (5). A criterion for the diagnosis, apart from arthritis symptoms, was a direct immunofluorescence (DIF) confirmed history of chlamydial genital tract infection (urethritis in men, cervicitis in women), which had occurred 1 to 3 months before the onset of arthritis symptoms. Twenty-eight patients were DIF-positive and 11 DIF-negative. The presence of antibodies against hsp60 were determined using an enzyme-linked immunosorbent assay (ELISA).

### Results

Mapping of B-cell epitopes has demonstrated that they consist of chlamydiae species and genus-specific epitopes, as well as epitopes cross-reacting with human hsp60 (6). In the present study IgG antibodies to unique and conserved synthetic peptide epitopes of chlamydial hsp60 were found in 7 (18%) and in 14 (35.9%) of the 39 SARA patients, respectively. All 7 samples with IgG antibody reactivity to peptide 151-162 also had antibodies to peptide 260-271. The sera with antibodies to 151-162 and 260-271 did not more often show MIF IgM or IgA antibody reactivity, or higher antibody titres to C. trachomatis of the Ig classes as compared to the hsp60-negative sera. However, only 4 samples were positive for IgA in MIF, which is too few cases to allow for any reliable conclusion. IgM antibodies were found in approximately 14% and 36% among the hsp-positive and -negative sera, respectively. Thus, half of the patients with antibodies to the hsp60 epitope that is conserved between chlamydiae and man did not have antibodies to a chlamydia-specific hsp60 epitope. This indicates that either the conserved epitope is more immunogenic than the unique epitope following a chlamydial infection or that some other microorganism might have been the initial trigger for the induction of antibodies in some SARA patients that are cross-reactive to the sequence conserved in the chlamydial and human hsp60 molecules. A positive DIF-test or the presence of neutralizing serum antibodies also were not correlated with the presence of hsp60 serum antibodies. Chlamydial antigen detection in the urethra and in the cervix did not correlate with the presence of chlamydial hsp60 antibodies in patients with ReA in a previous study (7), while in a second study the presence of antibodies to chlamydial hsp60 in patients with non-gonococcal urethritis were correlated with the isolation of C. trachomatis from the urethra (8). There was no difference between the chlamydial hsp60 antibody-positive and-negative samples in the time lag between the diagnosis of a C. trachomatis infection and the onset of SARA.

### Conclusion

Futher work will be necessary to convincingly establish that auto-antibody responses to hsps play a signigicant role in the immunopathology of chlamydial infection and its sequelae, e.g., SARA.

### References

1. Cerrone MC, Ma JJ, Stephens RS: Cloning and sequence of the gene for heat shock protein 60 from Chlamydia trachomatis and immunological reactivity of the protein. Infect Immunol 59:79–90, 1991.
2. Brunham RC, Peeling RW: Chlamydia trachomatis antigens: Role in immunity and pathogenesis. Infect Agents Dis 3:218–233, 1994.
3. Kihlström E, Grönberg A, Bengtsson A: Immunoblot analysis of antibody response to Chlamydia trachomatis in patients with reactive arthritis and ankylosing spondylitis. Scand J Rheumatol 18:377–383, 1989.
4. Oldstone MB: Molecular mimicry and autoimmune disease. Cell 50:819–820, 1987.
5. Bergström D, Domeika M, Vaitkiene D, Persson K, Mardh P-A: Prevalence of Chlamydia trachomatis, Chlamydia psittaci, and Chlamydia pneumoniae in blood donors and attendees of STD clinics. Clin Microbiol Infect a:253–260, 1996.
6. Yi Y, Zhong G, Brunham RC: Continuous B-cell epitopes in Chlamydia trachomatis heat shock protein 60. Infect Immunol 61:1117–1120, 1993.
7. Larsen B, Birkelund S, Mordhorst CH, Ejstrup L, Andersen LS, Christiansen G: The humoral immune response to Chlamydia trachomatis in patients with acute reactive arthritis. Br J Rheumatol 33:534–540, 1994.
8. Newhall WJ, Battieger B, Jones RB: Analysis of human serological response to proteins of Chlamydia trachomatis. Infect Immunol 38:1181–1189, 1982.
